# Computational modeling of the relationship between morphological heterogeneity and functional responses in mouse hippocampal astrocytes

**DOI:** 10.3389/fncel.2024.1474948

**Published:** 2024-10-17

**Authors:** Anna Freund, Alexander Mayr, Peter Winkler, Rene Weber, Aapo Tervonen, Ron Refaeli, Kerstin Lenk

**Affiliations:** ^1^Faculty of Computer Science and Biomedical Engineering, Institute of Neural Engineering, Graz University of Technology, Graz, Austria; ^2^Biosciences Unit, Faculty of Medicine and Health Technology, Tampere University, Tampere, Finland; ^3^Laboratory of Inbal Goshen, Hebrew University of Jerusalem, Edmond and Lily Safra Center (ELSC), Jerusalem, Israel; ^4^BioTechMed, Graz, Austria

**Keywords:** astrocyte, morphology, computational neuroscience, intracellular signaling, biophysical modeling, calcium dynamics

## Abstract

Recent studies indicate that astrocytes show heterogeneity in morphology and physiological function. They integrate synaptic signals and release calcium in reaction to active neurons. These calcium signals are not yet fully understood as they are highly dependent on the cell's morphology, which can vary across and within brain regions. We found structural heterogeneity among mouse hippocampal CA1 astrocytes based on geometric features, clustering 741 cells into six classes. Of those, we selected 84 cells and reconstructed their morphology based on confocal microscope images and converted them into multi-compartment models with a high detailedness. We applied a computational biophysical model simulating the intracellular ion and IP_3_ signaling and diffusion in those 3D cell geometries. The cells were stimulated with three different glutamate stimuli. Calcium mainly oscillated in the stimulated and the neighboring compartment but not in the soma. Significant differences were found in the peak width, mean prominence, and mean peak amplitude of the calcium signal when comparing the signals in the stimulated and neighboring compartments. Overall, this study highlights the influence of the complex morphology of astrocytes on intracellular ionic signaling.

## 1 Introduction

Over time, the conventional view that astrocytes primarily fulfill structural, metabolic, and regulatory roles has been challenged. Recent research spanning the past three decades has unveiled a more active role for astrocytes, indicating their involvement in modulating synaptic transmission and brain metabolism (Araque et al., 1999; Haydon and Carmignoto, 2006; Nedergaard and Verkhratsky, 2012). Each astrocyte consists of a soma with multiple outgoing branches that further divide into smaller branchlets, ultimately leading to the perisynaptic astrocyte processes (PAPs). Notably, the abundance and morphology of astrocytes exhibit significant variability across species, brain regions, and cortical layers (Zhou et al., 2019; Khakh and Deneen, 2019; Verkhratsky and Nedergaard, 2018; Holt, 2023; Baldwin et al., 2023). Comparative studies have revealed striking differences, such as human neocortical astrocytes being 2.6 times larger in diameter and possessing up to 10 times more primary branches than their rodent counterparts (Oberheim et al., 2009). Moreover, investigations by Lanjakornsiripan et al. (2018) have delineated distinct variations in cell orientation, territorial volume, and arborization among different layers within the somatosensory cortex of mice. Heterogeneity in astrocytes may also have an impact on brain functions and diseases. Grolla et al. (2013) suggested that calcium (Ca^2+^), an important cellular messenger, responses to Amyloid-β differ in primary cultured mouse astrocytes of the entorhinal cortex and the hippocampus. Exposure to Amyloid-β increased expression of the metabotropic glutamate receptor type 5 (mGluR5) and increased Ca^2+^ concentration in the hippocampus but not the entorhinal cortex. It may be the case that the lack of astrogliosis in the entorhinal cortex is a contributing factor to the higher vulnerability of this region to Alzheimer's disease.

Various studies have been conducted to classify astrocytes. Viana et al. (2023) statistically analyzed astrocytes from the mouse hippocampus, a region critical for learning and memory, and found structural heterogeneity in their morphology across hippocampal domains. They identified three types of astrocytes in the Cornu Ammonis area 1 (CA1) and two in the dentate gyrus. Lanjakornsiripan et al. (2018) performed hierarchical clustering on non-pial neocortical astrocytes based on morphological features and found four distinct classes. Batiuk et al. (2020) found five different astrocyte subtypes according to gene expression in the mouse cortex and hippocampus. Karpf et al. (2022) classified layer-specific astrocyte subtypes in the mouse dentate gyrus based on density measured with GFAP and SOX2 markers.

Astrocytes primarily communicate through Ca^2+^ transients, which can evolve as global events spanning over the entire astrocytic cell or localized responses within specific branches (Di Castro et al., 2011; Srinivasan et al., 2015; Semyanov et al., 2020). The mechanisms underlying intracellular Ca^2+^ dynamics can be categorized into at least two distinct pathways. The first pathway involves the binding of glutamate to metabotropic glutamate receptors (mGluRs) on the astrocytic plasma membrane, triggering the production of inositol 1,4,5-trisphosphate (IP_3_). Elevated IP_3_ levels increase the probability of opening the IP_3_ receptor (IP_3_R) channels at the endoplasmic reticulum (ER) membrane, leading to an influx of Ca^2+^ from the ER into the cytosol. This Ca^2+^ release can initiate a Ca^2+^-induced Ca^2+^ release (CICR) mechanism, further amplifying the cytosolic Ca^2+^ signal (Sharma and Vijayaraghavan, 2001). The SERCA (sarcoplasmic/endoplasmic reticulum Ca^2+^-ATPase) pump transports Ca^2+^ back from the cytosol to the ER. The second pathway involves the activation of glutamate transporters (EAAT1 and EAAT2 in the human brain and GLAST and GLT-1 as the rodent analogs) (Murphy-Royal et al., 2017; Mahmoud et al., 2019). The resulting changes in intracellular sodium (Na^+^) and potassium (K^+^) levels influence the activity of the Na^+^-Ca^2+^ exchanger (NCX) and the Na^+^-K^+^ ATPase (NKA). NCX can either export or import Ca^2+^, depending on the Na^+^ gradient, while NKA regulates the Na^+^ and K^+^ gradients across the plasma membrane (Kirischuk et al., 1997; Verkhratsky and Nedergaard, 2018). These described mechanisms highlight the complex interplay between glutamate signaling, ion homeostasis, and Ca^2+^ dynamics in astrocytes, underscoring their pivotal role in modulating neuronal activity and synaptic transmission (Araque et al., 2014).

However, heterogeneity in morphology may imply heterogeneity in function (Augusto-Oliveira et al., 2020), such as varying Ca^2+^ dynamics (Oberheim et al., 2012; Tsunematsu et al., 2021). In turn, Kruyer (2022) claims that heterogeneous Ca^2+^ dynamics influence morphological plasticity.

Computational models can aid in identifying the most relevant experiments to characterize and predict the function of a biological system (Lenk et al., 2024). Several papers include the morphology of astrocytes in their computational studies at various spatial scales. Verisokin et al. (2021) proposed an astrocyte network model with realistic, data-driven 2D cell morphologies. The model includes IP_3_-mediated Ca^2+^ signaling at the soma and branches as well as intra- and intercellular diffusion of IP_3_ and Ca^2+^. Although the model uses 2D cell templates, it can reproduce characteristic patterns of Ca^2+^ signaling to represent a single-plane imaging regime. Gordleeva et al. (2019) investigate with their neuron-astrocyte network model how Ca^2+^ dynamics in astrocytes can synchronize and coordinate neuronal network signaling. The model contains a neuron-astrocyte network of 100 synaptically-coupled neurons and two gap-junction-connected astrocytes. Each astrocyte consists of 53 compartments that are unit-length cylinders with a radius that reduces further away from the soma. The Ca^2+^ signaling is composed by IP_3_-mediated Ca^2+^ signaling and intracellular IP_3_ and Ca^2+^ diffusion. This model demonstrates that astrocytes serve as spatial and temporal integrators, responding to varying levels of neuronal activity with distinct Ca^2+^ dynamics that influence synaptic transmissions and facilitate spatial synchronization.

On the cellular level, a detailed multi-compartment astrocyte model has been introduced as ASTRO by Savtchenko et al. (2018). The astrocyte morphologies were reconstructed from two-photon excitation and correlational 3D electron microscopy images. The modeled dynamics included K^+^ fluxes, *IP*_3_ action and Ca^2+^ diffusion and buffering. On the subcellular level, a particle-based model of a PAP compartment was implemented by Denizot et al. (2019). Using their model, the authors were able to recreate stochastic Ca^2+^ signals and showed that their occurrence is heavily dependent on the spatial positioning of *IP*_3*R*_ channels.

However, no computational study is currently used to systematically investigate the relationship between astrocyte morphology and function. Our research question, therefore, is whether differences in the morphology influence ion dynamics in astrocytes. To answer the question, we will use an extended version of the Oschmann et al. (2017) model, including morphology and diffusion. Overall, astrocyte heterogeneity in both form and function has important implications for our understanding of brain function and diseases. By elucidating the specific roles of different astrocyte subtypes, we can gain insights into the complex networks that underlie brain function and identify potential targets for therapeutic intervention in neurological disorders.

## 2 Methods

### 2.1 Preprocessing of the astrocyte reconstructions

**Figure 1:**
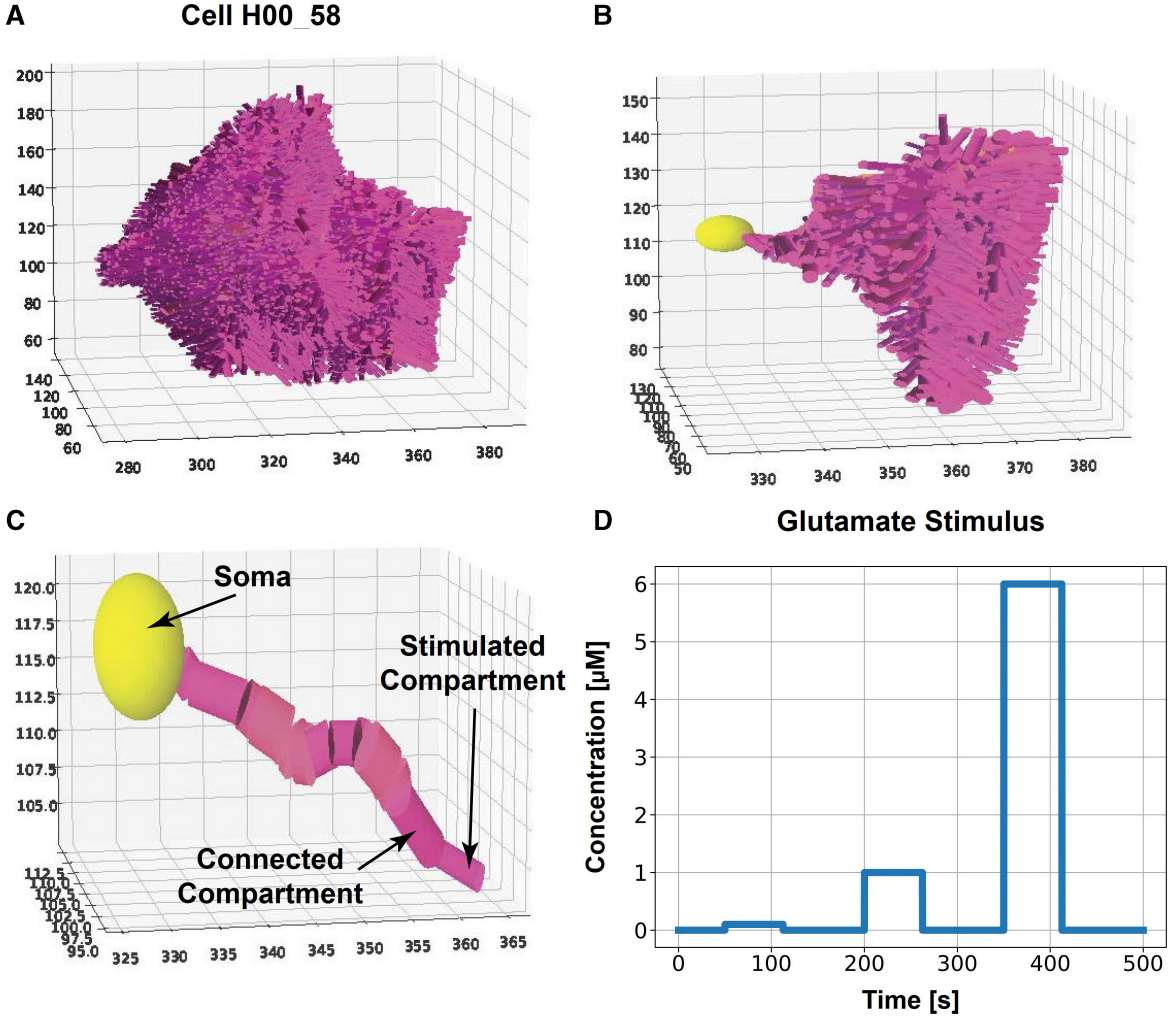
Compartmentalized astrocyte morphology model. The cell morphology, captured by a microscope image, is reconstructed by cylindrical consecutive compartments and a sphere for the soma. We stimulate the outermost compartment of a primary branch with glutamate and investigate the *Ca*^2+^ dynamics in one cell branch (primary branch with all subbranches) for all classes. **(A)** Example of a complete reconstructed astrocyte from class #3 and **(B)** the selected primary branch with side branches. **(C)** Selected primary branch [same as in **(B)**] with an indication of the stimulated compartment, connected compartment, and soma. The spatial scales in **(A–C)** is μ*m*. **(D)** Glutamate stimulus with concentrations of 0.1, 1, and 6 μ*M* for 62.5 *s* each over a total simulation time of 500 *s*.

The experimental data by Refaeli et al. (2021) was used to reconstruct the astrocyte morphologies (example of a reconstructed astrocyte in Figure 1A). As described in the paper, hippocampal CA1 tissue of male mice was made transparent by CLARITY, and astrocytes were labeled with GFAP::tdTomato and imaged with a laser scanning confocal microscope. Images of cubes with a size of 520 × 370 × 520 μ*m* *to* 635 × 635 × 1, 500 μ*m* were used to reconstruct single astrocytic domains with the filament tracer tool of the software *IMARIS* (Bitplane, UK) as described in Refaeli et al. (2021). We used six data sets from six different male mice for our analysis. The samples contained 1,765 astrocytes in total. The smallest recorded diameter of the branches was 0.75 μ*m*. All the work described in the following has been done with Python and MATLAB (see Supplementary Section 1.1 for details).

Naturally, the sample cubes include cropped astrocytes at the cube border region. We removed those cut cells with a threshold region of 2 μ*m* from the cube boundaries. Every cell with more than 20 branching points inside the region was rejected.

Then, 21 features, described in detail below and in Supplementary Section 1.2, were calculated from the remaining 866 cells. The features can be divided into four types: (1) being directly collected from the data, (2) from the minimum volume encapsulating ellipsoid (MVEE) calculation, (3) from the Sholl-like analysis, and (4) from the primary branches of the cell.

First, the *number of branching points* (when a branch divides into subbranches) and the *number of terminal points* of cell branches were directly taken from the datasets as features. Also, the cell radius was calculated using an iterative fitting algorithm based on the terminal points of the branches and a percentile threshold of 70%.

Second, for the calculation of the MVEE, the algorithm by Bowman and Heath (2023) was used, and the following eight features were derived: *Volume* of the ellipsoid that approximates the actual volume the astrocyte covers. *Roundness* and *sphericity* are measures for the cell morphology (Matías and Vallespí, 2014). *Aspect ratio* is the ratio between the largest and smallest ellipsoid axis comparable to the aspect ratio described by Baldwin et al. (2023) but considers a third dimension. The angles between the main axis are turned into features, denoted as *alpha, beta*, and *gamma*. Lastly, the feature called *main axis* is the length of the main axis of the ellipsoid.

Third, in the Sholl-like analysis (Sholl, 1953), we segmented the 3D cell area into shells with a distance of five μ*m* between each shell. Then, we calculated the number of branching points as a function of the radial distance from the soma center. The resulting curves are highly comparable to the original Sholl analysis (Sholl, 1953; Baldwin et al., 2023). From the Sholl-like analysis, we obtained the following four features (Baldwin et al., 2023): the *process maximum* denotes the maximum number of branching points in a shell; the *critical value* is the distance between the soma and the shell with the process maximum; the *primary branches* feature is the number of branches that start from the soma and, therefore, have a depth of one in the IMARIS dataset; the *maximum radius* is the maximum width of the shells to cover all terminal points of a cell.

Fourth, another important morphological feature is the characterization of the primary branches of a cell stemming directly from the soma. We calculated the mean, maximum, and standard deviation of the *diameter of the primary branches at the branching point*. The *primary angles* are the average of angles between the different primary branches. The mean, standard deviation, and maximum of the *branch length* are calculated using the branching points of each branch as start and endpoints. The maximum and mean *number of branching points from the primary branches* are calculated for each cell. The vector of 21 features is normalized using the *MinMaxScaler* function (from Python's scikit-learn).

### 2.2 Classification of the astrocyte morphologies

All cells with at least 100 branching points were selected for classification, reducing the dataset from 866 to 741. Two approaches for outlier detection are applied: Extreme Gradient Boosting (XGBoost) and anomaly detection with an Auto-Encoder (AE). Only features directly derived from the dataset without further calculation, i.e., branching points, terminal endpoints, maximum and mean diameter, were used.

XGBoost is an algorithm that combines the output of different outlier detection methods (Zhao and Hryniewicki, 2019). The framework performs different outlier detection methods, undergoes a selection step to choose the useful ones, and finally stacks the labels together and trains the classifier. We tried various available methods and selected Isolation Forest, Local Outlier Factor, and Elliptic Envelope. The implementation was done with functions provided by Python's scikit-learn bibliography.

The Auto-Encoder (AE) is a neural network with a bottleneck that forces compressed information (Chen et al., 2018; Hinton and Salakhutdinov, 2006) and is trained to reconstruct the input. Due to the bottleneck, only the prominent features are learned, and the network is expected to perform badly in reconstructing outliers, data points that deviate greatly from the majority. After the network finishes training, it is used to predict the training data again, which means inputting it and expecting it as the output. Loss was calculated to evaluate the performance of the network on each data point. Data points with a high loss were labeled as anomalies. The threshold of labeling a data point as an anomaly is set to the mean plus the standard deviation of the total loss. Outliers detected by both methods were removed, and 710 samples remained for further classification.

Two mathematical methods were used to find the optimal number of classes. The *knee locator* describes the maximum curvature of the explained variance as a function of the number of classes and is implemented in Python's kneed module. The *dendrogram* shows the Euclidian distance between joined clusters. The obtained number of classes from those two mathematical methods was used to classify with the following clustering algorithms implemented in Python's sklearn: *k*-means, Gaussian Mixture, and Agglomerative Clustering. Their classification results were compared with confusion matrices, showing how many different data points were put in the same class by those methods. We used three different methods to demonstrate the independence from the classification method. For the simulations, we used the class labels derived from *k*-means. *K*-means seemed to have the best overlap with the other two methods and was used for labeling the cells.

### 2.3 Statistical analysis of the morphological features

To investigate whether there are significant differences in the individual features between classes, we applied the non-parametric Kruskal-Wallis test for statistical differences in the medians between classes (in Python's sciPy package). For the *post-hoc* tests to determine which classes differ, we used Dunn's test from the scikit-posthocs library, applying the Dunn—Šidák correction for *p*-value adjustment.

### 2.4 Cell selection for simulations

For the simulations of Ca^2+^ dynamics in astrocytes (see next section), cell morphologies from each class and dataset were selected. For the cell morphology, we created a compartmentalized model made out of cylinders. Therefore, the *x, y*, and *z* coordinates of two consecutive points in one branch of the IMARIS dataset with their corresponding mean diameter were used to construct a cylinder. A sphere with the same diameter as the branch with the largest diameter was used to approximate the cell's soma. Due to the high computational cost, we selected not the whole astrocyte but a primary branch with 15 and 25 compartments to make the simulation results comparable between cells (Figures 1B, C). A primary branch is defined as consecutive compartments starting from the soma. At branching points, the primary branch continued along the branch with the largest diameter. We included all subbranches of that chosen primary branch (Figure 1C). The outermost compartment was stimulated (see next section).

### 2.5 Simulations of the astrocytic calcium dynamics

For simulating the intra- and extracellular ion and messenger signals, we used the computational model introduced by Oschmann et al. (2017), which includes two pathways for intracellular Ca^2+^ signals in astrocytes: (1) the Ca^2+^ release from the internal Ca^2+^ stores in the ER and (2) Ca^2+^ entering through the plasma membrane. An extracellular glutamate stimulus activates both pathways. The activity of these two pathways is highly dependent on the distance from the soma, as the surface-to-volume ratio increases as the branches become thinner, which occurs with higher distance. Thus, the volume ratio of internal Ca^2+^ stores to the intracellular space decreases. Ca^2+^ buffering is lumped together with the intracellular Ca^2+^ concentration (Oschmann et al., 2017; De Pittà et al., 2009).

We applied the following two modifications to the model. Firstly, the current fluxes between cytosol and ER were removed, and we only consider charge fluxes between intra- and extracellular space when computing membrane voltage *V* over time (Farr and David, 2011; Witthoft and Em Karniadakis, 2012):


(1) $$\begin{array}{l} \frac{dV}{dt}=-\frac{1}{C_{m}}\left(I_{NCX}-2I_{GluT}+I_{NKA}+I_{Na_{leak}}+I_{K_{leak}}\right). \end{array}$$

The membrane capacitance is denoted as *C*_*m*_ and the currents of the NCX, EAAT1/2, and NKA as *I*_*NCX*_, *I*_*GluT*_, and *I*_*NKA*_, respectively. The leak currents of Na^+^ and K^+^ are defined as *I*_*N*_*a*__*leak*__ *I*_*K*_*leak*__, respectively. Secondly, the valence of Ca^2+^, which is two, was taken into account for calculating the intracellular Ca^2+^ concentration, ${\left[Ca^{2+}\right]}_{i}$ (Luo and Rudy, 1994):


(2) $$\begin{array}{r} \frac{d{\left[Ca^{2+}\right]}_{i}}{dt}=\frac{1}{2}\cdot \frac{A}{F\cdot Vol}I_{NCX}+\frac{A}{F\cdot Vol}\sqrt{ratio_{ER}}\\ \times \left(I_{IP_{3}R}-I_{Serca}+I_{C_{ER}leak}\right). \end{array}$$

The area of the outer cell membrane is denoted by *A*, the Faraday constant by *F*, the volume by *Vol*, and the area of the internal Ca^2+^ store by $A\cdot \sqrt{ratio_{ER}}$. The currents of the IP_3_R, SERCA pump and Ca^2+^ leak from the ER are defined as *I*_*I*_*P*__3_*R*_, *I*_*Serca*_, and *I*_*C*_*ER*_*leak*_, respectively.

The model by Oschmann et al. (2017) was designed as a single-compartment model without diffusion along the branch. We used the multi-compartment approach suggested by Gordleeva et al. (2019). The connections between compartments can be set with a connection matrix with values of either 0 for no connection or 1 for a set connection. The diffusion of the ions and IP_3_ between compartments in the three areas ER, cytosol, and extracellular space was integrated based on Kang and Othmer (2009) (see Table 1 for the diffusion coefficients).

**Table 1:** Diffusion coefficients for cytosol, endoplasmic reticulum (ER), and extracellular space (ECS) used in the multi-compartment model.

**Parameter**	**Value**	**Unit**	**Description of diffusion coefficient**	**References**
D_*IP*3_	3e-10	$\frac{m^{2}}{s}$	IP_3_ in the cytosol	Kang and Othmer, 2009
D_*Ca*_	3e-11	$\frac{m^{2}}{s}$	Ca^2+^ in the cytosol	Kang and Othmer, 2009
D_*Na*_	1.33e-9	$\frac{m^{2}}{s}$	Na^+^ in the cytosol	Qian and Sejnowski, 1989
D_*K*_	1.96e-9	$\frac{m^{2}}{s}$	K^+^ in the cytosol	Qian and Sejnowski, 1989
D_*CaER*_	3e-11	$\frac{m^{2}}{s}$	Ca^2+^ in the ER	Kang and Othmer, 2009
D_*CaES*_	1.3e-11	$\frac{m^{2}}{s}$	Ca^2+^ in the ECS	
D_*NaES*_	1.33e-9	$\frac{m^{2}}{s}$	Na^+^ in the ECS	Qian and Sejnowski, 1989
D_*KES*_	1.96e-9	$\frac{m^{2}}{s}$	K^+^ in the ECS	Qian and Sejnowski, 1989

A neuronal glutamate stimulus with concentrations of 0.1, 1, and 6 μ*M* (as described in De Pittà et al., 2009 and Oschmann et al., 2017) was applied to the tip of the selected primary branch (Figures 1C, D). The stimulus was applied for 62.5 s, respectively, and the total simulation time was 500 s. We used the same rectangular stimulus described in De Pittà et al. (2009) to allow comparability. We used an implicit Runge-Kutta method (Hairer and Wanner, 1996) as a numerical integration method for the non-linear differential equations with a time step *dt* = 1*ms*.

### 2.6 Ion dynamic analysis

To evaluate the physiological function, we extracted features measuring the Ca^2+^ signal transmission from the stimulated compartment to the connected compartment and to the soma, respectively. We did this for each of the six classes and the three stimulation intensities (0.1, 1, and 6 μ*M* glutamate) separately. Firstly, we calculated the *changes in the peak width* when the Ca^2+^ signal diffused from the stimulated compartment to the connected compartment using the *findpeak* function in MATLAB. Also, the *change of peak prominence*, also obtained by the *findpeak* function, was evaluated. Furthermore, we investigated the *change in mean peak amplitude* between the three selected compartments. Secondly, we calculated the differences between the maximum signal amplitude between the three compartments. We applied again the non-parametric Kruskal-Wallis with the Dunn-Šidák correction to test for statistical differences.

Additionally, we analyzed the influence of the morphology categorized into the six different classes using a three-way ANOVA (analysis of variance) with the three factors – stimulus intensity, class, and compartment. Therefore, we calculated the number of peaks using the *findpeak* function with the constrain of a minimum peak prominence of 30% of the maximum Ca^2+^ concentration in the respective compartment and a maximum peak width of 15 s. For the simulated intracellular Na^+^ concentrations, we calculated the maxima for each stimulus intensity, class, and compartment. For the intracellular K^+^ concentrations, we calculated the respective minima.

## 3 Results

### 3.1 Feature extraction and classification reveals differences in CA1 astrocyte morphology

**Figure 2:**
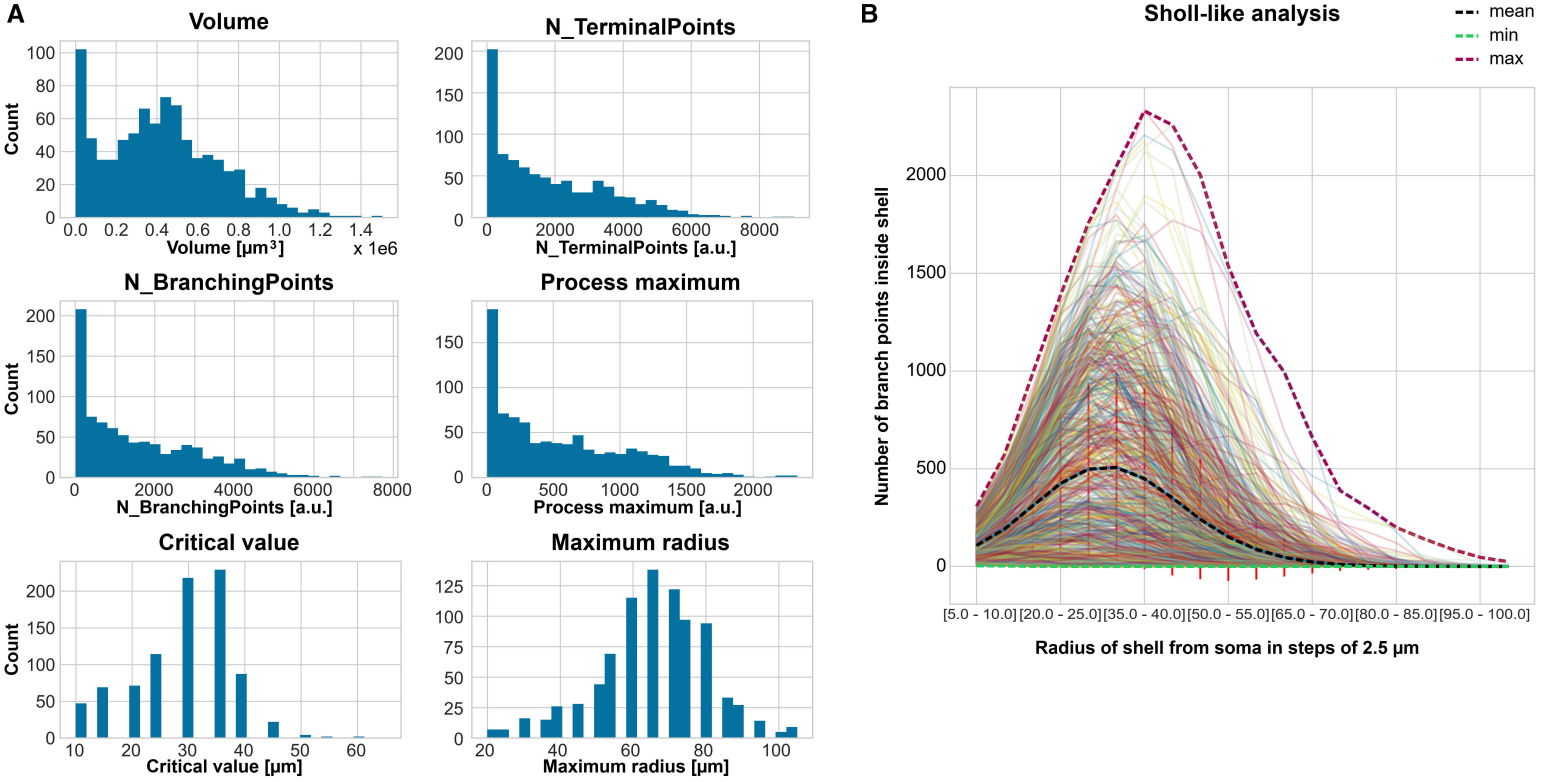
Feature extraction to describe the astrocyte morphology for the classification. **(A)** Count histogram of six representative out of 21 features for all 866 cells. **(B)** Sholl-like analysis of all cells indicating the minimum, mean, and maximum of the number of branch points per shell.

We calculated 21 features to describe the morphology of the 866 cells. Their histograms are displayed in Supplementary Figure S2, of which six are selected for (Figure 2A). Most cells have high counts for small volume, number of terminal points, number of branching points, and process maximum. Thus, we applied the outlier detection (see Section 2.2) before the classification. Besides that, most of the cells had a *volume* around 0.4 μ*m*^3^, around 3,400 *terminal points*, around 250 *branching points*, and a *process maximum* around 80. The *critical value* had the highest count of cells at 36 μ*m* and the *maximum radius* at 64 μ*m*. The Sholl-like analysis provided a mean *process maximum* of 500, a mean *number of primary branches* of 5 μ*m*, a mean *critical value* of 30 μ*m*, and a mean *maximum radius* of around 70 μ*m* (Figure 2B).

**Figure 3:**
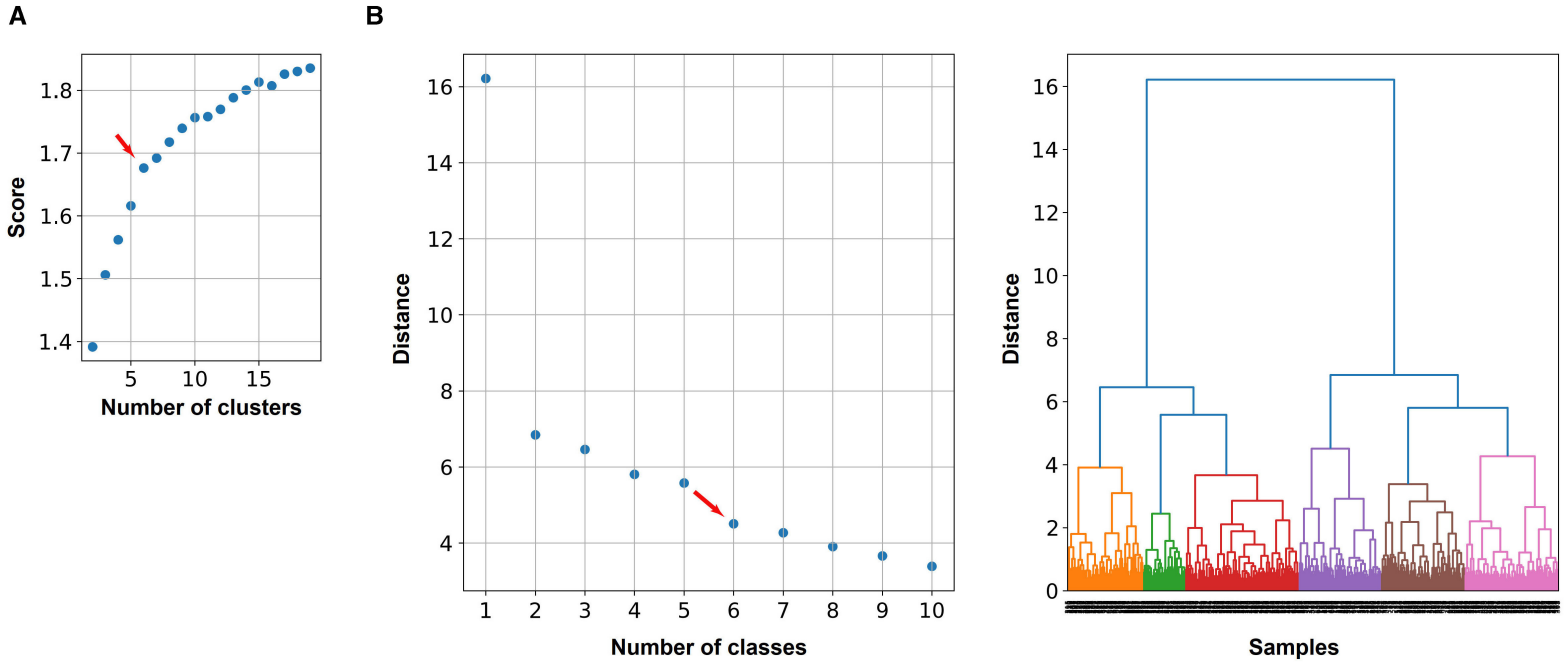
Two methods were used to determine the optimal number of classes to distinguish astrocyte morphologies: the knee locator and the Euclidean distance between joined clusters. **(A)** Explained variance that is used to find the knee (maximum curvature) indicated by the red arrow. The knee locator is based on k-means clustering, whereby for each number of classes, the score (explained variance) is calculated. **(B)** The dendrogram shows how much the Euclidian distance (on the *y*-axis) increases by joining two classes. This is based on the hierarchical clustering. The red arrow indicates a clear decline in the distance between classes 5 and 6.

**Figure 4:**
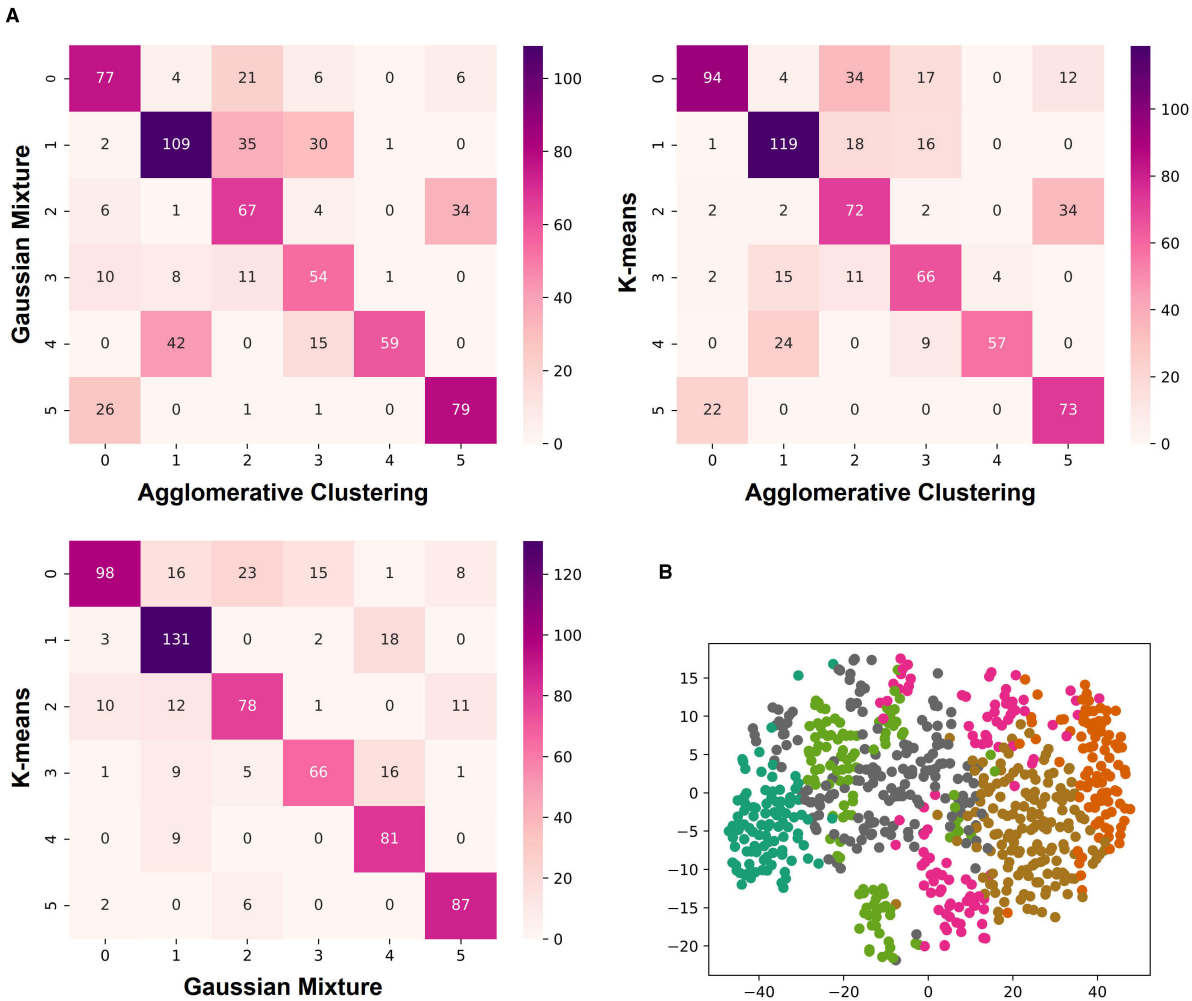
Three different algorithms clustered the morphological features of mouse CA1 astrocytes into six classes. **(A)** Confusion matrices comparing the labeled classes of the Gaussian Mixture model, k-means, and Agglomerative Clustering. There is a row *x* and a column *y* for each label. The number in the matrix gives the number of labels identified as class *x* by algorithm A as class *y* by algorithm B, with *x*,*y* = 0, 1, 2, 3, 4, 5. **(B)** Labeled data points by *k*-means as a t-SNE plot.

**Figure 5:**
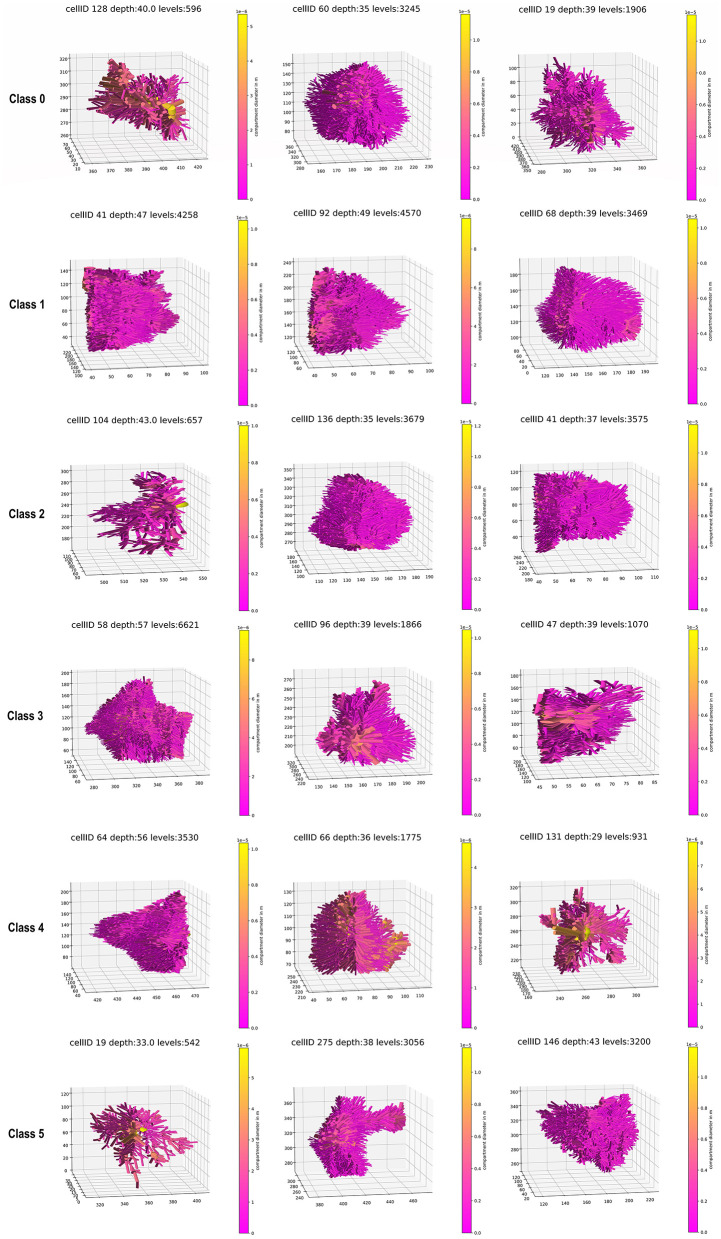
Three examples of a whole astrocyte for each class.

To define the number of classes for the classification, the *knee locator* divided the dataset into six different classes (Figure 3A). This was coherent with the *dendrogram*, which showed a clear increase of the Euclidian distance between joined clusters when going from 6 to 5 classes (Figure 3B). The high numbers on the diagonals of the confusion matrices when comparing the classification results by k-means, Gaussian Mixture model, and Agglomerative Clustering indicated that the majority of the cells were classified with the same label (Figure 4A). The labels derived from k-means were used for the final classification. Figure 4B displays the labeled data points by k-means plotted as t-distributed Stochastic Neighbor Embedding (t-SNE). Figure 5 shows three example cells from each of the six classes. We confirm that the obtained classes do not cluster according to the initial datasets (Table 2).

**Table 2:** Occurrence of each data label (class number) in each data set.

**Occurrence**	**C10**	**H00**	**H01**	**H02**	**H04**	**C11**	**Sum**
Class 0	60	4	11	8	38	40	161
Class 1	0	44	52	22	28	8	154
Class 2	35	11	15	7	23	21	112
Class 3	16	19	1	3	11	48	98
Class 4	0	37	27	7	16	3	90
Class 5	47	0	6	1	24	17	95
Outlier	5	11	8	2	3	2	31
Sum	163	126	120	50	143	139	741

**Figure 6:**
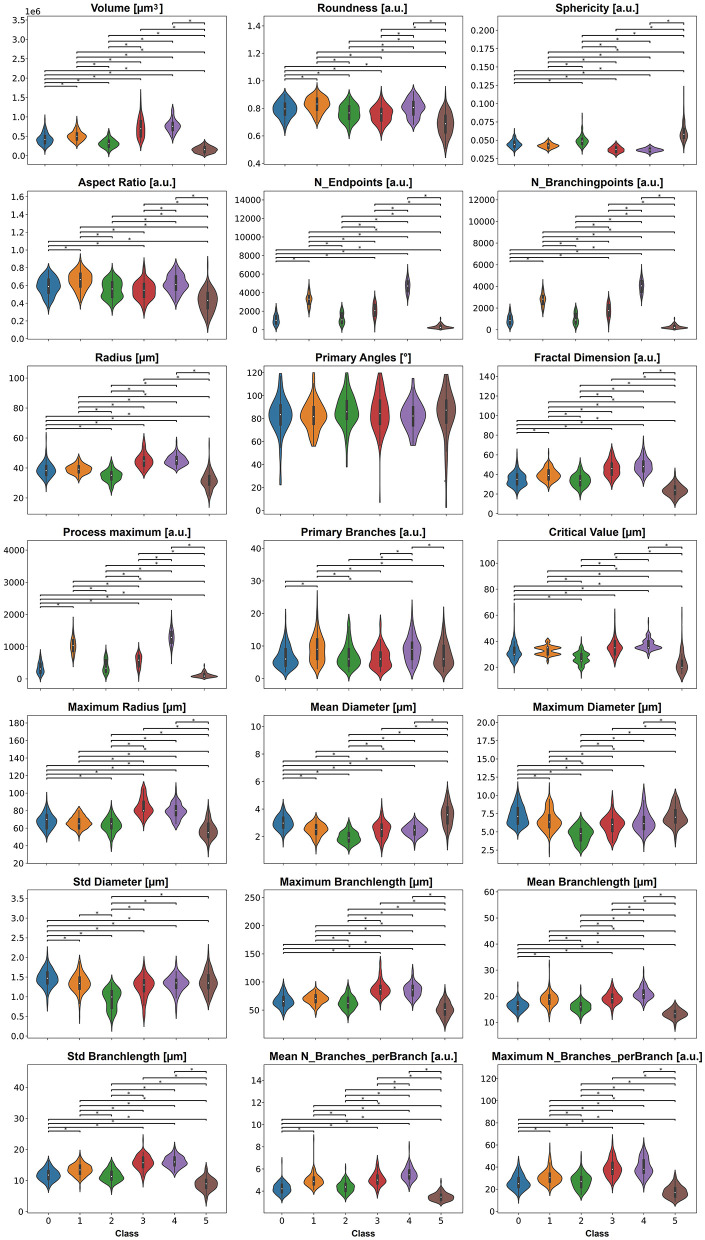
The 21 features, except primary angles, display significant differences between the six classes. *P*-values smaller than 0.05 are indicated with a star.

We tested all features for differences between classes using the Kruskal-Wallis test with a *p*-value < 0.05 (Figure 6); the exact *p*-values are stated in Supplementary Table S3. The violin plots indicate that all features besides feature *Primary Angles* have significant differences between most of the classes.

### 3.2 Calcium signal partially depends on morphology

**Figure 7:**
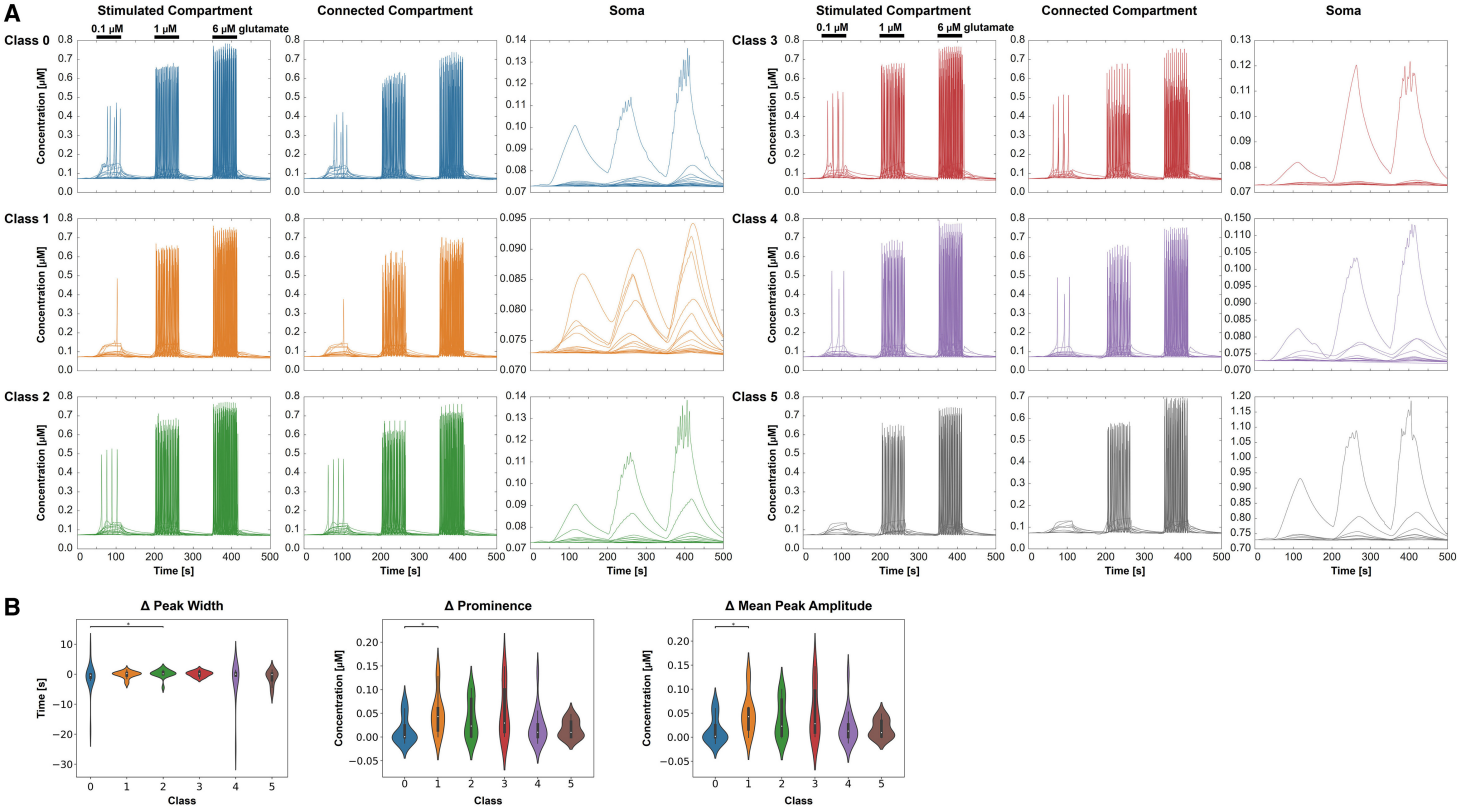
Intracellular Ca^2+^ behavior in the three selected compartments for each class (0–5), respectively. **(A)** Ca^2+^ concentrations over time for the different classes and compartments. For clear visibility of the Ca^2+^ signals, the *y*-axis may change for each condition. The stimulation periods have been indicated above the signals in the stimulated compartment. **(B)** Comparing the extracted features—change in the mean peak width, change in the peak prominence, and change in mean peak amplitude—from the Ca^2+^ dynamics for the six classes. All three features are based on a signal change from the stimulated to the connected compartment.

Figure 7A displays the intracellular Ca^2+^ concentration over time for the six classes and the three different compartments. As expected, we observed an increase of the Ca^2+^ concentration with stimulus intensity and a decrease from the stimulated compartment toward the some. Oscillations occurred mainly in the stimulated and the connected compartment. As for most cells, there is no proper oscillation visible in the soma, we compared the *change in peak width* over groups only from the stimulated compartment to the connected compartment (Figure 7B). A significant difference was found between classes 0 and 2. The comparison of the *change in mean peak prominence* from the stimulated compartment to the connected compartment indicated significant results for classes 0 and 1 (Figure 7B). Significant differences between classes 0 and 1 were also detected for the *change of the mean peak amplitude* from the stimulated compartment and the connected compartment (Figure 7B). The three-way ANOVA with the three factors—stimulus intensity, class, and compartment—revealed pairwise differences between classes 0 and 1, 0 and 2, 0 and 4, 0 and 5, and 1 and 3 (p < 0.05) for the connected compartment and stimulus intensity of 0.1 μ*M*.

### 3.3 Potassium and sodium dynamics showed no differences between classes

**Figure 8:**
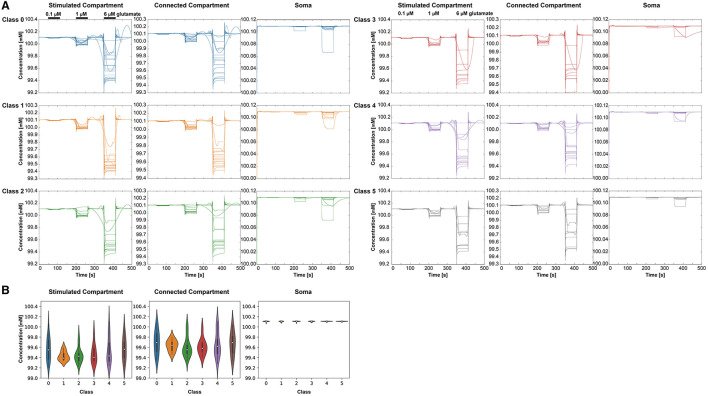
Intracellular K^+^ behavior in the three selected compartments for each class (0–5), respectively. **(A)** K^+^ concentrations over time for the different classes and compartments. For clear visibility of the K^+^ signals, the *y*-axis may change for each condition. The stimulation periods have been indicated above the signals in the stimulated compartment. **(B)** Violin plots showing the minima of the K^+^ amplitudes at 6 μ*M* stimulus for the different classes and the three compartments—stimulated compartment, connected compartment, and soma.

**Figure 9:**
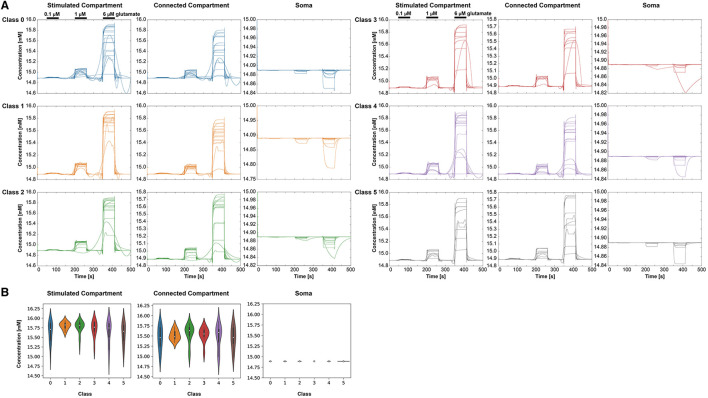
Intracellular Na^+^ behavior in the three selected compartments for each class (0–5), respectively. **(A)** Na^+^ concentrations over time for the different classes and compartments. For clear visibility of the Na^+^ signals, the *y*-axis may change for each condition. The stimulation periods have been indicated above the signals in the stimulated compartment. **(B)** Violin plots showing the minima of the Na^+^ amplitudes at 6 μ*M* stimulus for the different classes and the three compartments—stimulated compartment, connected compartment, and soma.

With increasing glutamate stimulus, the intracellular K^+^ concentration was reduced (Figure 8A). The lowest concentrations were measured in the stimulated compartment. The Na^+^ concentration decreased in the soma with increasing stimulus, whereas it increased in the other two compartments (Figure 9A). The Kruskal-Wallis test showed no significant differences between the classes (Figures 8B, 9B).

## 4 Discussion

Our research explores the relationship between astrocyte morphology and function, specifically focusing on how structural differences influence ion dynamics. Astrocytes exhibit significant morphological heterogeneity across species and brain regions (Zhou et al., 2019; Baldwin et al., 2023; Torres-Ceja and Olsen, 2022).

GFAP is expressed in astrocytes, and antibodies against GFAP are a standard marker for immunolabeling the astrocyte's intermediate filament. In Refaeli et al. (2021), all astrocytes were tagged and marked using the GFAP promoter through viral infection. Consequently, it is likely that these astrocytes were in a certain state of activation due to both GFAP gene expression and the viral injection into the brain. However, this cannot be proven, as in the hippocampus (unlike the cortex), most astrocytes express GFAP even without being activated (Chai et al., 2017).

Cells can be categorized based on molecular (Karpf et al., 2022) and morphological characteristics (Lanjakornsiripan et al., 2018; Viana et al., 2023). For example, Viana et al. (2023) found structural heterogeneity in the hippocampus subregions CA1 and dentate gyrus by testing for statistical differences in morphological features like total process length, number of processes, Sholl analysis, and last intersection radius. Most of the calculated geometrical features that we used for the classification have been used in other studies as well (Baldwin et al., 2023; Herde et al., 2020; Savtchenko et al., 2018; Viana et al., 2023). Our classification revealed six classes of astrocytes within the CA1 region. Determining the number of classes is quite challenging since there is no ground truth. Thus, to provide a solid ground, we trained three different clustering algorithms, aiming to obtain similar classification results. K-means, Gaussian mixture model, and Hierarchical clustering yielded an overall coherent classification.

The cylindrical compartments, reconstructed from the microscope images, allow the use of their surface and volume data for the differential equations of our model. So far, only the ASTRO model by Savtchenko et al. (2018) comes with such a high detailedness of the 3D geometry as we have it in our computational model.

Fine astrocytic processes are responsible for the majority of astrocytic Ca^2+^ signals (Bindocci et al., 2017). Morphological variations may have functional implications (Molotkov et al., 2013; Grolla et al., 2013). Astrocytic Ca^2+^ signaling, a primary mode of communication, involves complex mechanisms, including glutamate-triggered intracellular Ca^2+^ (Semyanov et al., 2020). While computational models have been developed to study astrocyte function at various scales (Lenk et al., 2024; Manninen et al., 2018), a systematic investigation of the relationship between morphology and ion dynamics is lacking. We created a computational model, based on the work of Oschmann et al. (2017) and Gordleeva et al. (2019), including 3D morphology and intra- and extracellular diffusion. Our multi-compartment model is derived from confocal microscope images of the CA1 region (Refaeli et al., 2021). This model enables analyzing mechanisms of Ca^2+^ release from the internal Ca^2+^ stores in the ER and Ca^2+^ entering through the plasma membrane, both depending directly or indirectly on extracellular glutamate released by neurons.

The simulated *Ca*^2+^ dynamics (Figure 7) vary across the three conditions: the class based on morphological features, stimulus intensity, and compartment type. While stimulated and connected compartments indicate similar signals, the respective signals in the soma do not display clear oscillations for most cells. The glutamate stimulus of 1 and 6 μ*M* leads to oscillatory behavior as also demonstrated by De Pittà et al. (2009), while the 0.1 μ*M* glutamate stimulus only induces in some classes a few *Ca*^2+^ peaks. Astrocytes in class 5 exhibit, for example, a smaller volume and fractal dimension, resulting in a lack of *Ca*^2+^ peaks with a low glutamate stimulus. However, astrocytes in class 1 with a high roundness value, high process maximum, and a high mean number of branches can exhibit a small number of *Ca*^2+^ peaks with a low glutamate stimulus.

Zur Nieden and Deitmer (2006) studied *Ca*^2+^ dynamics in hippocampal rat astrocytes and obtained similar results with a 10 μ*M* glutamate stimulus as we did when stimulating our model with 6 μ*M* glutamate. They report that the stimulus only elicited oscillations in 63% of the astrocytes, which might be an indication of heterogeneity. Similar to our simulations, Nett et al. (2002) measured the *Ca*^2+^ oscillations and their diffusion across four primary processes of a mouse hippocampal astrocyte.

The results for *Na*^+^ are comparable with Rose and Karus (2013). The authors obtained comparable *Na*^+^ concentrations of around 13 and 18 *mM* when stimulating with 1 and 10 μ*M* glutamate, respectively. We observed no significant differences in *K*^+^ and *Na*^+^ ion dynamics between the six classes, unlike with *Ca*^2+^. This may result from the fact that the *Ca*^2+^ dynamics in the model are partly dependent on the intracellular stores in the ER and the IP_3_R channels, whose role in the dynamics is highly dependent on the local branch morphology. *K*^+^ and *Na*^+^ dynamics, on the other hand, only depend on the current through the plasma membrane. As for the diffusion, it is modeled similarly between the ion species and, therefore, does not lead to differences between their dynamics.

Using the cell information such as position and shape can be used in the future to reconstruct and simulate a whole cube with connected astrocytes (Refaeli et al., 2021; Ma et al., 2016). As a starting point, the work by Verisokin et al. (2021) could be used in which the authors constructed a 2D spatial astrocyte network to model Ca^2+^ signals in a multicellular construction. Furthermore, studying the influence of ion dynamics on the astrocyte morphology (Denizot et al., 2019), which we did not consider here, is an interesting aspect for future work. Overall, astrocytes are glial cells that play an important role in brain activity. Combining imaging and computational modeling techniques can help obtain more information on astrocyte morphological and functional complexity.

## Data Availability

The datasets presented in this study can be found in online repositories. The names of the repository/repositories and accession number(s) can be found below: https://github.com/kerstinlenk/AstrocyteHeterogeneity.
