# Supplementary material for: Computational modeling of the relationship between morphological heterogeneity and functional responses in mouse hippocampal astrocytes
Source: Front Cell Neurosci. 2024 Oct 17;18:1474948. doi: 10.3389/fncel.2024.1474948 (PMC11524972; doi:10.3389/fncel.2024.1474948)
Supplement: Supplementary file 1 [file Data_Sheet_1.PDF]

Supplementary Material

1 SUPPLEMENTARY DESCRIPTIONS

1.1 Software versions

- For the feature calculation, we used the Python version v3.10.1, NumPy v1.22.4, Pandas v1.3.5, matplotlib v3.5.1, pymatreader v0.0.30, scikit-learn v1.3.0, and SciPy v1.7.3.
- For the classification, we used Python version v3.9.13, NumPy v1.23.5, Pandas v1.4.4, matplotlib v3.5.2, scikit-learn v1.0.2., tensorflow v2.12.0, xgboost v2.0.2, and SciPy v1.13.1. Python is freely available.
- For the statistics and the violin plots, we used scikit_posthocs v0.9.0, seaborn v0.11.2. For the calcium peak detection and the calculation of the minima and maxima of the K^+ and Na^+ concentrations, respectively, we used MATLAB version R2019b (developed by MathWorks). The three-way ANOVA was also done in MATLAB.

1.2 Preprocessing for the feature vector

1.2.1 Initial datasets

The dataset comprises two principal components: fine points data and branching points data.

Fine Points Data: This component encompasses the x, y, and z coordinates of each mapped point within the imaged astrocyte. Additionally, it includes a file that details the connections between these points, referenced by their respective indexes.

Branching Points Data: This component includes the points where the astrocyte's processes bifurcate into two distinct branches, as well as the soma and branch terminal points, collectively referred to as branching points (BPs). Each branching point is accompanied by comprehensive information, including:

- **Spatial information**: The spatial position of the BP.
- **Diameter**: The diameter of the branch measured at the BP.
- **Category**: A classification of the branching points into three types: Soma, dendrite branch, and dendrite terminal.
- **Depth**: The number of BPs preceding the current one, counted from the soma's direction.
- **Type**: A categorization of the branching points into Soma, dendrite branch, and dendrite terminal.
- **Filament ID**: The identification number of the filament to which the BP belongs.
- **ID**: The unique identification number of the current BP.

1.2.2 Edge cell removal

The initial dataset includes cells that underwent segmentation due to the preparation process, particularly affecting cells situated at the periphery of the sample cube, leading to incomplete depiction (Refaeli et al., 2021). Such cells introduce a potential bias to the statistical analysis. To address this, an algorithm was developed using a threshold method along with the positional data of all cells in the dataset to effectively filter out the cells that were cut off at the edge of the sample cube.

Firstly, the dimensions of the cube were computed based on the minimum and maximum Fine Point coordinates in the x, y, and z directions within the dataset. Subsequently, a threshold of 2 μm was subtracted from these results to establish a reduced-size cube. The subtraction of the smaller cube from the larger one delineates a threshold area of 2 μm (Figure S1), wherein cells are susceptible to being truncated during the preparation process. Then all the points of the dataset inside the threshold area were counted for every cell. Ultimately cells with more than 20 BPs in the threshold area of the brain slice were discarded as incomplete. To summarize, the border threshold and the number of points within this threshold were configured to exclude all cells at the edge of the cube safely. The possibility that nearly fully intact cells might also be excluded was embraced in favor of prioritizing security.

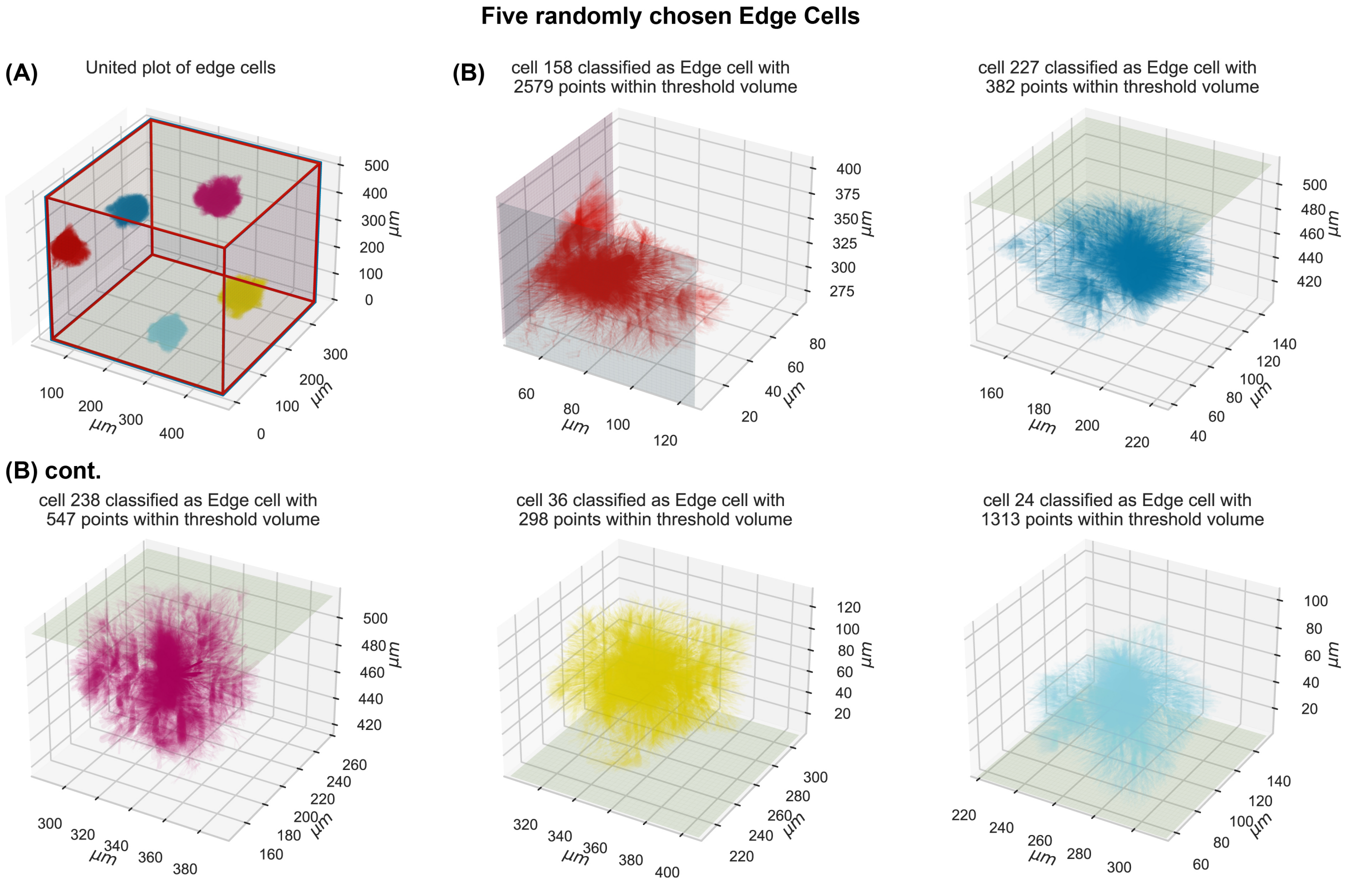

Figure S1: Removal of cell at the edge of the sample cube. **(A)** Sample cube with red and blue outlines. **(B)** View is zoomed in on one corner of the cube, respectively, to visualize the gap representing the threshold volume being 2 μm from the outer border (blue) in all directions. Fine Position points within this volume are counted. A cell containing more than 20 points in the threshold area is labeled as an edge cell and discarded.

1.3 Features calculated directly from the dataset

Two features were calculated directly from the dataset, the average branch diameter (*av_Branchdiameter*) and the number of endpoints (*N_Endpoints*). The average branch diameter of each cell is obtained by using the mean of the diameter values at all the branching points of each cell from the branching point data frame and calculating their average value. To calculate the number of endpoints, the BPs labeled as "Dendrite Terminal" are counted for each cell.

1.4 Minimum Volume Encapsulating Ellipse (MVEE)

As a generalizing way to evaluate the 3D morphology of individual cells, the cells were encapsulated in an ellipsoid using the least volume possible. To achieve this, the Minimum Volume Enclosing Ellipsoid (MVEE) algorithm was used (Perren, 2015). In short, the MVEE algorithm itself is a classical optimization problem that calculates the standard form of an ellipsoid returning the matrix **A** and the position of the center c_{Ell}. This method, however, runs into time constraint problems for large datasets (Perren, 2015), which is even more of an issue for 3D data, as this extension further increases the computation time needed. Here, to alleviate these problems, only the dendrite terminal points of each cell are used for the MVEE calculation. This process is shown in Figure 4.4 in Matías and Vallespí (2014).

From the resulting ellipsoid matrix, the length of the axis and the rotation can be extracted using singular value decomposition on the positive definite ellipsoid matrix **A**. This calculation uses the SciPy v1.7.3 package for Python (Virtanen et al., 2020). From these values, the features of interest were calculated. These are the main ellipsoid angles *alpha*, *beta*, and *gamma*, the *length of the main axis*, the *volume of the ellipsoid*, as well as the *roundness* and *sphericity indexes*. The results are then stored for every cell and compiled into the feature vector for the classification task.

1.4.1 Ellipsoid Volume (Volume)

The volume of the ellipsoid was calculated with the ellipsoid axes a, b, and c taken from the encapsulating ellipsoid produced by:

$$V_{Ell} = \frac{4}{3} \cdot \pi \cdot a \cdot b \cdot c \tag{S1}$$

Ellipsoid Sphericity According to Matías and Vallespí (2014), *sphericity* is a measure of the morphological properties of a particle, or in this case, a cell, that is most dependent on the elongation property. The sphericity ratio or index Ψ is calculated with the equation

$$\Psi = \frac{\mathbf{S}_n}{\mathbf{S}} \quad , \tag{S2}$$

where $\mathbf{S}_n$ is the surface area of the encapsulating ellipsoid and $\mathbf{S}$ is the surface area of a sphere occupying the same volume as the ellipsoid. This index takes on values between 0 and 1, with spheres having a ratio of 1 (Matías and Vallespí, 2014).

Ellipsoid Roundness In contrast to the sphericity, the *roundness* parameter is dependent on the sharpness of the corners of the object. As this thesis involves ellipsoids, there are three pairs of corners in every sample cell, and from their radii, the roundness ($\mathcal{R}$) of the ellipsoid can be calculated with the equation (Matías and Vallespí, 2014):

$$\mathcal{R} = \frac{\frac{1}{n}\sum_{i=1}^{n} r_i}{r_{max}}. \tag{S3}$$

1.5 Sholl-like Analysis

Sholl analysis is a prevalent technique in neurobiology for quantifying the complexity of dendritic arbors (Binley et al., 2014). It involves generating a so-called Sholl profile by plotting the number of dendrite

intersections as a function of the radial distance from the soma center (Baldwin et al., 2023). This profile provides valuable insights into dendritic complexity.

In this manuscript, a Sholl-like profile was calculated using 50 linearly spaced values with a step size of 5 μm as the radii of the spheres. The number of branching points within each cell is counted using the Euclidean distance of the branching points from the cell soma. The points are then summed up and attributed to the shells in correspondence to this distance. This method yields a curve comparable to the classical Sholl analysis.

Finally, this is iterated over all the cells of the dataset. From the Sholl analysis, we obtained the following features (Baldwin et al., 2023): The *process maximum* denotes the maximum number of branching points in a shell. The *critical value* is the distance between the soma and the shell with the process maximum. The *primary branches* feature is the number of branches that start from the soma and, therefore, have a depth of 1 in the IMARIS dataset. The *maximum radius* is the maximum width of the shells.

1.6 Data collected from the Primary Branches

Another important morphological feature is the characterization of the primary branches of a cell stemming directly from the soma region. For this, only branches with a starting depth of less than five were considered. For these branches, the following features were calculated.

The *mean*, *maximum*, and *standard deviation of the diameter of the primary branches* based on the IMARIS branching point data, and the *mean*, *maximum*, and *standard deviation of the length of the primary branches* by calculation of the Euclidean distance between the branching points of each branch.

By summing up the number of branching points in each branch a measure for the branching complexity of the main branches is obtained. The mean and max of this value are taken as cell features.

Before starting the outlier detection, we used Min-Max scaling to normalize the data.

1.7 Outlier Detection

The network architecture is a sequential model consisting of 7 dense layers, each with a dropout rate of 0.1. The dropout is used to stabilize the network and avoid over-fitting.

- Activation function: ReLu
- Output activation function: Sigmoid
- Layer sizes: 32/16/8/4/16/32/25 neurons
- Loss: Mean Squared Logarithmic Error
- Evaluation Metric: Mean Squared Error
- Optimizer: Adam
- Training-set Validation-set split: 80% - 20%
- Epochs: 70
- Batch Size: 512
- Total parameters: 2981 (trained with 25 features)

1.7.1 Isolation Forest

The Isolation Forest algorithm returns the anomaly scores of each data point with recursive partitioning. Isolation Forest is a method from the `Ensemble Methods` module. We used all default parameter settings for the classifier.

1.7.2 Local Outlier Factor

The local outlier factor measures the density deviation to the data point neighbors. The number of neighbors was set to 100 with the keyword argument `n_neighbors`. This is a method from the `Nearest Neighbors` module.

1.7.3 Elliptic Envelope

This method classifies outliers based on covariance estimation and is from the *scikit* module `Covariance Estimators`. The keyword argument for contamination was set to 0.095, which is the expected proportion of outliers in the dataset. The other parameters were left with the default settings.

1.8 Classification

For the Gaussian Mixture model, the keyword argument `random_state` was set to zero, and for the Agglomerative Clustering algorithm, the `ward` method was used. For k-means classification, all keyword arguments were left in the default setting.

2 SUPPLEMENTARY TABLES AND FIGURES

2.1 Tables

Classes<br>Features	0-1	0-2	0-3	0-4	0-5
1 - Volume	0.0019621	4.71881e-05	2.16185e-15	3.24565e-23	3.80544e-21
2 - Roundness	0.00019093	0.0291145	0.000524351	0.995012	1.77675e-19
3 - Sphericity	0.0621814	0.000126189	8.75662e-19	1.2872e-22	4.90258e-19
4 - Aspect ratio	9.20616e-06	0.182108	0.0282811	0.488524	2.71122e-18
5 - N_Endpoints	1.22489e-37	0.998715	4.82741e-08	1.55247e-53	3.40231e-09
6 - N_Branchingpoints	1.17402e-37	0.999111	5.8462e-08	1.99433e-53	2.88269e-09
7 - Radius	0.982489	1.26404e-07	1.2464e-15	4.21057e-18	1.93433e-14
8 - Primary Angles	1	0.315687	0.978694	0.999994	0.774608
9 - Fractal Dimension	4.85856e-06	0.999909	2.9345e-18	2.70447e-25	3.24652e-14
10 - Process Maximum	2.64279e-42	0.857855	3.32306e-05	1.16071e-47	1.90114e-08
11 - Primary Branches	1.29978e-07	0.999941	1	1.68175e-05	0.98714
12 - Critical Value	0.754294	6.60148e-09	0.000403204	4.31088e-11	6.22913e-17
13 - Max. radius	0.988518	0.00714044	6.7637e-22	3.74437e-13	7.46521e-11
14 - Mean Diameter	2.49063e-11	1.31432e-39	1.28364e-08	8.3268e-10	0.00780197
15 - Max. Diameter	0.00120066	1.22593e-41	1.65175e-09	0.000113277	1
16 - Std. Diameter	0.000164456	1.64016e-46	1.50369e-08	0.000436649	0.015269
17 - Max. Branchlength	0.148833	0.902867	9.9212e-26	7.35362e-20	2.32818e-09
18 - Mean Branchlength	2.37122e-12	0.966054	6.33586e-14	2.79824e-28	6.26656e-12
19 - Std. Branchlength	3.14347e-07	0.999373	2.74383e-25	1.87174e-28	1.07277e-10
20 - Mean N_branches/branch	5.109e-19	0.888068	8.09557e-19	5.07374e-36	4.31066e-10
21 - Max. Branches/branch	4.61063e-06	0.998125	1.86496e-24	2.22999e-25	1.41181e-10

Table S1: P-values for each feature and all class combinations.

Classes<br>Features	1-2	1-3	1-4	1-5	2-3
1 - Volume	8.97715e-15	1.80647e-05	1.03054e-10	3.95526e-37	5.96683e-31
2 - Roundness	3.13981e-11	3.84377e-14	0.106462	1.57874e-37	0.99277
3 - Sphericity	3.65386e-11	7.19319e-10	6.61327e-13	6.05019e-30	2.523e-34
4 - Aspect ratio	4.46673e-11	1.66221e-12	0.319826	6.10487e-39	0.99997
5 - N_Endpoints	1.42916e-26	5.8025e-07	0.000176188	9.30591e-68	4.59349e-05
6 - N_Branchingpoints	1.00459e-26	4.77927e-07	0.000191898	5.67613e-68	4.66485e-05
7 - Radius	1.7843e-10	7.61866e-12	4.25664e-14	5.23368e-18	1.53915e-36
8 - Primary Angles	0.121648	0.831327	1	0.465645	0.999274
9 - Fractal Dimension	1.20975e-06	0.000110554	7.40819e-09	1.74969e-33	3.23289e-18
10 - Process Maximum	4.74193e-27	2.59611e-12	0.0689228	5.31689e-71	0.0387325
11 - Primary Branches	9.29585e-05	2.15891e-06	1	0.00187394	0.999424
12 - Critical Value	1.69442e-13	0.104692	6.22805e-07	1.12314e-22	5.65539e-20
13 - Max. radius	0.202404	5.41551e-26	2.34154e-16	6.51674e-08	1.97249e-33
14 - Mean Diameter	1.17776e-10	1	0.999999	2.13555e-20	7.76964e-09
15 - Max. Diameter	2.07072e-22	0.045568	0.992018	0.0221783	7.25752e-09
16 - Std. Diameter	4.46475e-24	0.329674	1	0.999998	6 .42975e-12
17 - Max. Branchlength	0.00249768	5.55562e-16	1.15119e-11	1.80767e-16	3.56233e-28
18 - Mean Branchlength	2.52098e-14	0.948084	1.37453e-05	9.60403e-41	6.43855e-16
19 - Std. Branchlength	4.25072e-08	2.17999e-07	1.6468e-09	3.90776e-30	3.31467e-25
20 - Mean N_branches/branch	9.1494e-11	0.994655	2.14248e-05	8.66757e-47	1.26485e-11
21 - Max. Branches/branch	0.00317067	5.08093e-08	6.97691e-09	7.57219e-28	1.32817e-17

Table S2: P-values for each feature and all class combinations.

Classes<br>Features	2-4	2-5	3-4	3-5	4-5
1 - Volume	1.63208e-40	1.42551e-05	0.555808	5.02906e-57	1.15839e-68
2 - Roundness	0.0038003	5.05245e-08	7.29601e-05	4.0955e-05	1.0547e-18
3 - Sphericity	9.85814e-39	5.95545e-05	0.995148	2.23333e-59	2.29118e-64
4 - Aspect ratio	0.000848428	9.20862e-09	8.36209e-05	1.34197e-06	3.00558e-21
5 - N_Endpoints	1.88154e-41	3.28871e-10	1.34076e-17	8.29835e-27	1.2093e-83
6 - N_Branchingpoints	1.62656e-41	3.33913e-10	1.19046e-17	8.78946e-27	1.03257e-83
7 - Radius	1.74014e-39	0.256098	0.999797	4.59769e-47	4.22226e-50
8 - Primary Angles	0.171102	1	0.827252	1	0.502067
9 - Fractal Dimension	8.66668e-25	2.95754e-10	0.76398	1.1443e-50	5.30998e-60
10 - Process Maximum	1.18202e-33	3.95454e-11	1.52158e-18	6.24937e-21	4.35366e-75
11 - Primary Branches	0.00138665	1	6.27621e-05	0.972538	0.0120999
12 - Critical Value	1.46591e-31	0.15313	0.127042	1.39034e-29	1.29079e-42
13 - Max. radius	6.58194e-23	0.0132085	0.665158	9.75434e-50	7.30253e-37
14 - Mean Diameter	4.84585e-07	8.86907e-50	0.999999	1.47583e-16	7.21427e-18
15 - Max. Diameter	1.28715e-13	2.18702e-31	0.807271	4.75918e-07	0.00216284
16 - Std. Diameter	4.07038e-17	2.71595e-21	0.829505	0.177308	0.999454
17 - Max. Branchlength	1.86798e-22	5.04219e-05	0.998318	1.01736e-51	1.16019e-43
18 - Mean Branchlength	7.24579e-30	3.24152e-07	0.0159181	2.41452e-40	1.08184e-59
19 - Std. Branchlength	3.10818e-28	3.2723e-07	0.999482	6.59217e-54	1.89929e-57
20 - Mean N_branches/branch	7.47719e-25	1.06082e-12	0.00827139	3.9804e-44	1.57618e-65
21 - Max. Branches/branch	1.49158e-18	1.21418e-11	1	1.39178e-52	2.95118e-53

Table S3: P-values for each feature and all class combinations.

Class	Classified datasets	Sum datasets
0	C10_128, C10_216, C10_357, C11_176, C11_19, C11_42, H00_257, H01_213, H01_255, H02_143, H02_155, H02_60, H02_87, H04_101, H04_112, H04_127, H04_63	17
1	C11_76, H00_127, H00_146, H00_161, H00_168, H00_172, H00_41, H00_71, H00_73, H00_86, H01_105, H01_121, H01_92, H01_94, H02_147, H02_68, H02_81	17
2	C10_104, C10_246, C11_105, C11_111, C11_144, H00_106, H00_154, H00_200, H00_78, H02_41, H02_82, H04_125, H04_136, H04_50, H04_78	15
3	C10_142, C10_177, C11_47, C11_63, C11_64, C11_92, H00_137, H00_210, H00_58, H04_96	10
4	H00_141, H00_147, H00_214, H00_219, H00_299, H00_64, H01_116, H01_119, H01_130, H01_164, H01_168, H01_184, H01_66, H02_43, H02_76, H04_131	16
5	C10_19, C10_201, C10_232, C10_388, C10_68, C11_146, C11_232, C11_275, H01_275	9

Table S4: The dataset plus ID of the cells used for simulations for each class.

2.2 Figures

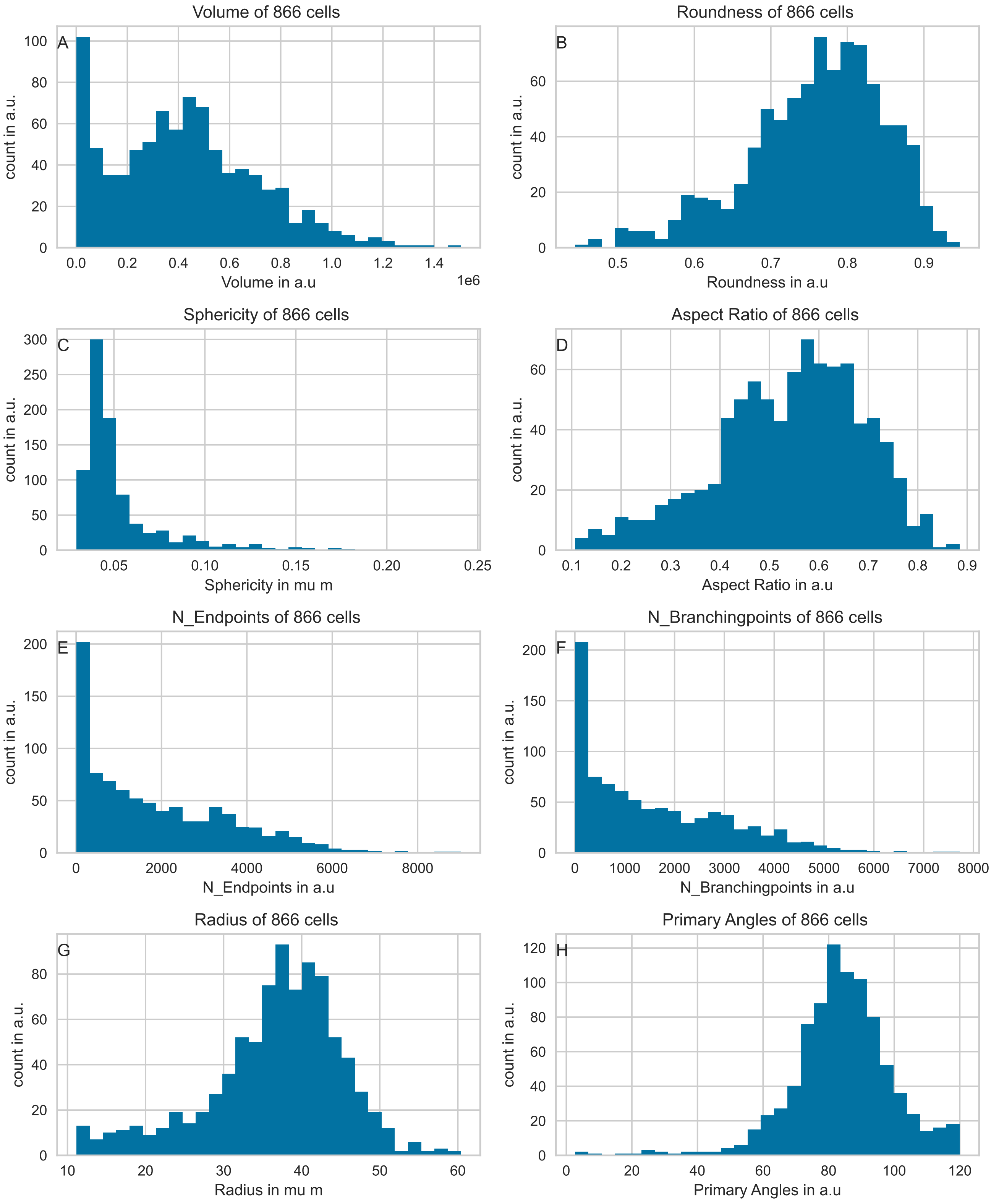
Volume of 866 cells
A
count in a.u.
Volume in a.u
1e6
Roundness of 866 cells
B
count in a.u.
Roundness in a.u
Sphericity of 866 cells
C
count in a.u.
Sphericity in mu m
Aspect Ratio of 866 cells
D
count in a.u.
Aspect Ratio in a.u
N_Endpoints of 866 cells
E
count in a.u.
N_Endpoints in a.u
N_Branchingpoints of 866 cells
F
count in a.u.
N_Branchingpoints in a.u
Radius of 866 cells
G
count in a.u.
Radius in mu m
Primary Angles of 866 cells
H
count in a.u.
Primary Angles in a.u

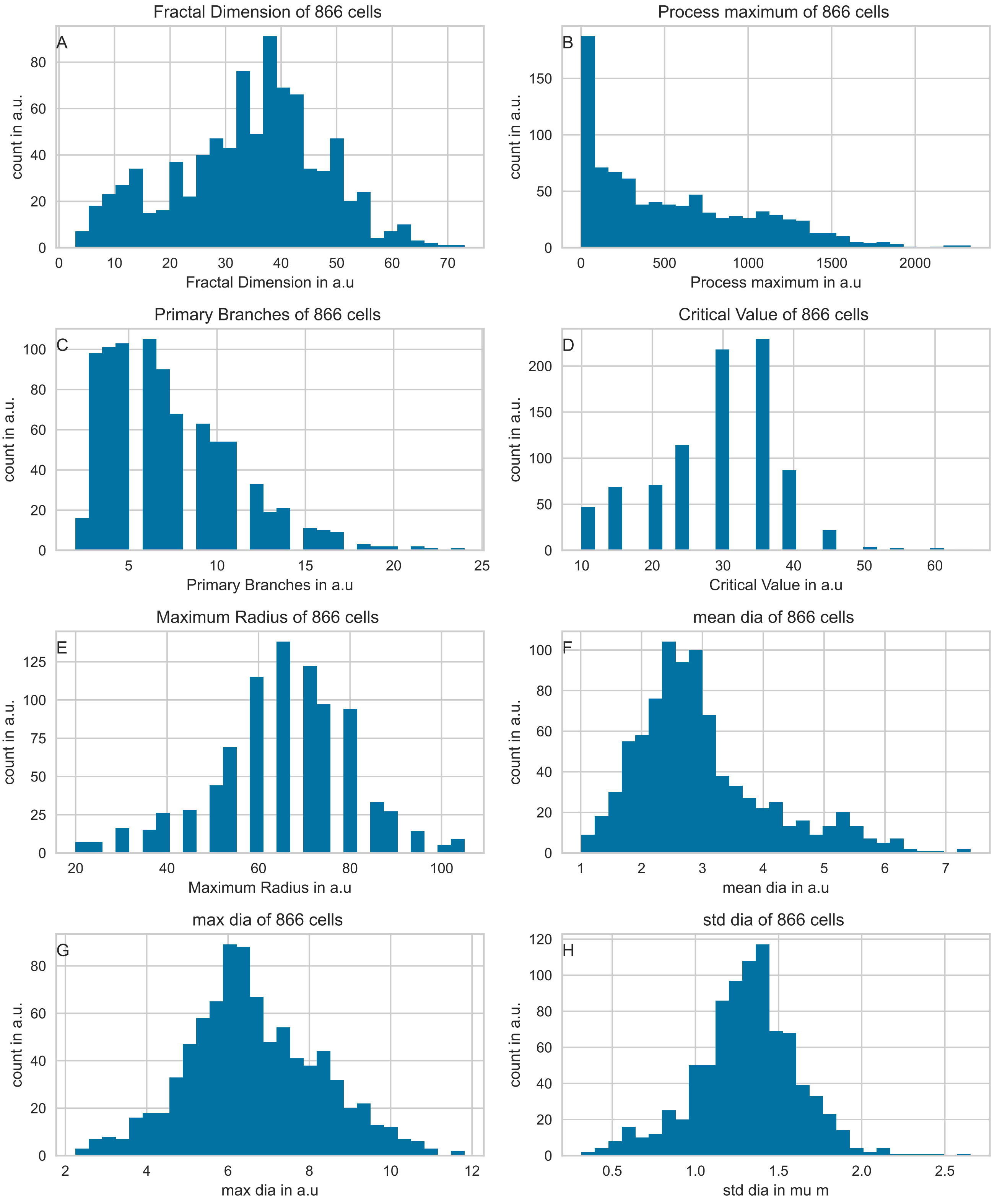
Fractal Dimension of 866 cells
A
count in a.u.
Fractal Dimension in a.u
Process maximum of 866 cells
B
count in a.u.
Process maximum in a.u
Primary Branches of 866 cells
C
count in a.u.
Primary Branches in a.u
Critical Value of 866 cells
D
count in a.u.
Critical Value in a.u
Maximum Radius of 866 cells
E
count in a.u.
Maximum Radius in a.u
mean dia of 866 cells
F
count in a.u.
mean dia in a.u
max dia of 866 cells
G
count in a.u.
max dia in a.u
std dia of 866 cells
H
count in a.u.
std dia in mu m

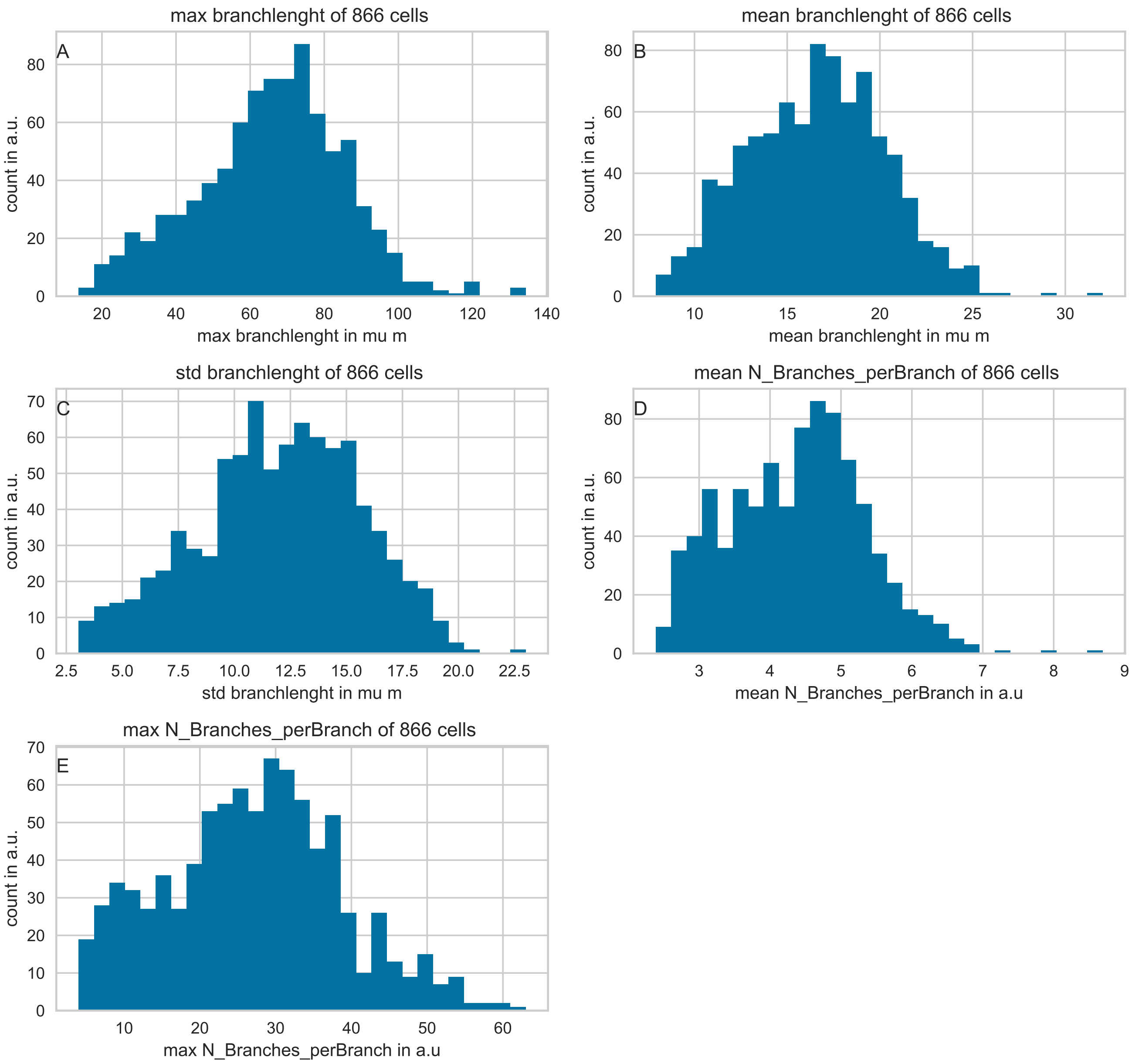

Figure S2: Histograms for all 21 features.

REFERENCES

Baldwin, K. T., Murai, K. K., and Khakh, B. S. (2023). Astrocyte morphology. *Trends in Cell Biology* doi:10.1016/j.tcb.2023.09.006

Binley, K. E., Ng, W. S., Tribble, J. R., Song, B., and Morgan, J. E. (2014). Sholl analysis: a quantitative comparison of semi-automated methods. *Journal of neuroscience methods* 225, 65–70

Matías, I. A. C. and Vallespí, M. D. A. (2014). Orientation, sphericity and roundness evaluation of particles using alternative 3d representations

[Dataset] Perren, G. (2015). Generate an ellipse through the MVEE method. `https://gist.github.com/Gabriel-p/4ddd31422a88e7cdf953`

Refaeli, R., Doron, A., Benmelech-Chovav, A., Groysman, M., Kreisel, T., Loewenstein, Y., et al. (2021). Features of hippocampal astrocytic domains and their spatial relation to excitatory and inhibitory neurons 69, 2378–2390. doi:10.1002/glia.24044

Virtanen, P., Gommers, R., Oliphant, T. E., Haberland, M., Reddy, T., Cournapeau, D., et al. (2020). SciPy 1.0: Fundamental Algorithms for Scientific Computing in Python. *Nature Methods* 17, 261–272. doi:10.1038/s41592-019-0686-2
